# Non-Destructive Prediction of Titratable Acidity and Taste Index Properties of Gala Apple Using Combination of Different Hybrids ANN and PLSR-Model Based Spectral Data

**DOI:** 10.3390/plants9121718

**Published:** 2020-12-06

**Authors:** Vali Rasooli Sharabiani, Sajad Sabzi, Razieh Pourdarbani, Edgardo Solis-Carmona, Mario Hernández-Hernández, José Luis Hernández-Hernández

**Affiliations:** 1 Department of Biosystem Engineering, Faculty of Agriculture and Natural Resources, University of Mohaghegh, Ardabili 56199-11367, Iran; s.sabzi@uma.ac.ir (S.S.); r_pourdarbani@uma.ac.ir (R.P.); 2 Faculty of Engineering, Autonomous University of Guerrero, Chilpancingo 39087, Mexico; 09302@uagro.mx (E.S.-C.); mhernandezh@uagro.mx (M.H.-H.); 3 Division of Research and Graduate Studies, TecNM/Technological Institute of Chilpancingo, Chilpancingo 39070, Mexico

**Keywords:** spectroscopy, hybrid ANN, non-destructive estimation, apple, PLSR, wavelengths

## Abstract

Non-destructive estimation of the internal properties of fruits and vegetables is very important, because better management can be provided for subsequent operations. Researchers and scientists around the world are focusing on non-destructive methods because if they are developed and commercialized, there will be an impressive change in the food industry. In this regard, this paper aims to present a non-destructive method based on Vis-NIR spectral data. The different stages of the proposed algorithm are: (1) Collection of samples of Gala apples, (2) Spectral data extraction by spectroscopy, (3) Pre-processing of spectral data, (4) Measurement of chemical properties of titratable acidity (TA) and taste index, (5) Selection of key wavelengths using hybrid artificial neural network-firefly algorithm (ANN-FA), (6) Non-destructive estimation of the properties using two methods of hybrid ANN- Particle swarm optimization algorithm and partial least squares regression. For considering the reliability of methods for estimating the chemical properties, the prediction operation was executed in 300 iterations. The results represented that the mean and standard deviation of the correlation coefficient and the root mean square error of hybrid ANN-PSO and PLSR for TA were 0.9095 ± 0.0175, 0.0598 ± 0.0064, 0.834 ± 0.0313 and 0.0761 ± 0.0061 respectively. These values for taste index were 0.918 ± 0.02, 3.2 ± 0.39, 0.836 ± 0.033 and 4.09 ± 0.403, respectively. Therefore, it can be concluded that the hybrid ANN-PSO has a better performance for non-destructive prediction of the two mentioned chemical properties than the PLSR method. In general, the proposed method can predict the chemical properties of TA and taste index non-destructively, which is very useful for mechanized harvesting and management of post-harvest operation.

## 1. Introduction

Apples belong to the Rosaceae family. The origin of apples is reported to be Asia, especially the Republic of Kazakhstan according to Forsline et al. [1] Apples are grown all over the world in temperate, subtropical and tropical climates. Today, due to the growing demand for food products, the need for automation in agriculture is becoming more apparent. On the other hand, many developed countries such as Japan and the European Union have set strict standards for the quality and health of imported food products. Therefore, in order to gain more share in the global export market, steps should be taken in order to adopt new developments in post-harvest technology [2,3].

Examination and grading of fruits is one of the post-harvest operations that has received more attention due to the growth of demand for healthy and high quality products [4,5]. In the last four decades, various techniques such as X-ray, optical, ultrasonic, electromagnetic, and infrared spectroscopy have been used for non-destructive evaluation of fruits; these are highly desirable and faster and more economical than destructive methods [6,7]. Among the mentioned techniques, the infrared spectroscopy has become more practical due to its high accuracy. Numerous studies have been accomplished on the use of Vis-NIR spectroscopy to predict chemical properties of fruit such as oranges [8], tangerines [9], grapes [10], pears [11], cherries [12], apricots [13], avocados [14], bananas [15], peaches [16], and tomatoes [17]. Moreover, studies on the maturity evaluation of agricultural products have been reported by several researchers [18,19,20].

Nicolai et al. [21] have made a comprehensive review on the application of NIR spectroscopy for determining the quality of fruits and vegetables. Fan et al. [22] developed a portable spectroscopy system at the range of 200–1000 nm to estimate the amount of soluble solid contents (SSC). Their system consisted of an optical probe, a spectrometer, a microcontroller, and a power supply. Light is transmitted to the fruit from a halogen source (12 V and 25 W) by a fiber optic ring. A detector received light that penetrated into the fruit tissue and was transmitted to the spectrometer. Fuji apples were placed one by one on the probe and their spectral features were collected. A model was constructed for SSC using partial least squares (PLS) with a coefficient of determination, root mean square error (RMSE) and standard deviation ratios of 0.777, 0.561 and 2.114, respectively. Li et al. [11] developed multi-cultivar and individual-cultivar models for prediction of SSC. The results showed that the multi-cultivar model was superior to individual-cultivar model; and the competitive adaptive reweighted sampling (CARS) was better than the Monte Carlo-uninformative variable elimination (MC-UVE) and the successive projections algorithm (SPA). To select the effective variables, the multi-cultivar models of CARS-PLS and CARS-MLR performed an effective prediction of SSC of three pear cultivars with the same accuracy. Generally, the results showed that the multi-cultivar model was good at prediction of SSC, the CARS was a powerful tool for selecting effective variables, and CARS-PLS and CARSMLR were excellent for spectral calibration. Mogollona et al. [23] studied internal physical abnormalities of mango (soft core and brown flesh) at the final stages of maturity using Vis-NIR spectroscopy at the range of 200–1000 nm. The number of 141 mango samples was collected at harvest time and 30 days after harvest at 12 °C. The results showed that wavelengths of 650–550 nm were able to detect the internal physical abnormality of mango. But they could not make a diagnosis of the type of anomaly. They only identified healthy and unhealthy fruit. Healthy fruits had higher reflection intensities than fruits with internal abnormalities in both storage and harvesting. Lan et al. [24] evaluated quality properties of apple and apple puree such as texture/rheology, SSC, acidity, dry matter content and insoluble solids. The PLS models had a good ability to estimate different properties based on the coefficient of determination (R2), e.g., the coefficients of determination for viscosity, cell wall content, dry matter, SSC and TA were R2 > 0.82, R2 > 0.81, R2 > 0.83, R2 > 0.80 and R2 > 0.80. Jamshidi et al. [25] evaluated the feasibility of predicting the chemical properties of Valencia oranges (such as SSC to TA and BrimA ratios) using Vis-NIR spectroscopy. Combinations of moving average and Savitzky–Golay smoothing filters, standard normal variant (SNV) and Multiplicative scatter correction (MSC) were used, and models were developed based on partial least squares (PLS) and principle component regression (PCR). The mean square root of the prediction of PLS model for SSC, TA, SSC/TA and BrimA were 0.33, 0.7, 1.03 and 0.37, respectively; and the correlation coefficients were 0.96, 0.86, 0.87 and 0.92, respectively. They also suggested the BrimA as the best indicator of fruit flavor. Escribano et al. [26] developed a model for fast and non-destructive evaluation of soluble solids (SSC) and dry matter content (DMC) of sweet cherries at two different temperatures of 0 and 23 °C at the range of 729 and 975 nm. The coefficient of determination (R2) of the model for SSC and DMC was 0.922–0.946 and 0.910–0.933, respectively.

As can be observed, various research has been conducted on the usage of non-destructive methods for detecting the different properties of fruits. In this regard, this study presents a non-destructive algorithm based on two hybrids of artificial neural network hybrids, namely ANN-FA and ANN-PSO as well as a partial least squares regression method using spectral data to estimate the chemical properties of the titratable acidity (TA) and the taste index of the Gala cultivar.

## 2. Materials and Methods

**Figure 1:**
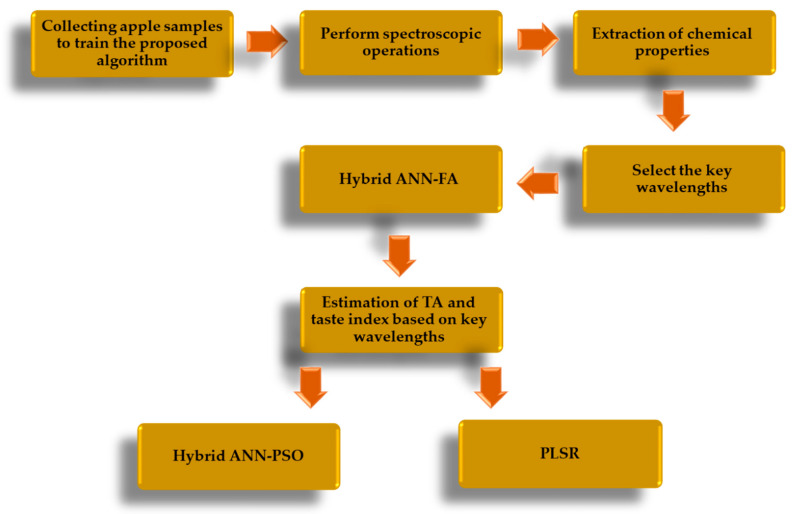
Flowchart of different stages of training the proposed algorithm for non-destructive estimation of chemical properties of titration acidity and taste index of Gala cultivar.

Figure 1 demonstrates the flowchart of the different stages of algorithm proposed for non-destructive estimation of the chemical properties of TA and taste index. In this study, hybrid ANN-FA was used to select key wavelengths and the hybrid ANN-PSO and PLSR were used to predict the mentioned properties.

### 2.1. Data Collection

In order to train the proposed algorithm, Gala apple samples were collected from orchards of the Kermanshah province. In general, 140 days after flowering, Gala apples are ripe and ready to be harvested. After consulting with different gardeners, the ripening time of Gala cultivar apples was determined based on their experience during the past few years. Then the total numbers of 150 samples were collected during 3 stages, namely 20 days before the ripening time, 10 days before the ripening time and at the time of ripening. In fact there were 50 fruits for each phase. At each stage, after collecting the samples, they were transported to the laboratory to extract spectral properties and to measure chemical properties.

### 2.2. Extraction of Spectral Properties from Samples

#### 2.2.1. Hardware of System Used

In order to extract spectral data, a hardware system was used that has different components. These components are:

- Laptop with Intel Core i3 CFI specifications, 330 M at 2.13 GHz, 4 GB of RAM, Windows 10. Spectra Wiz software was installed on the laptop to record spectral data of each sample.
- EPP200NIR spectrometer (StellarNet, Tampa, FL, USA) equipped with indium-gallium-arsenide detector. The spectral range of this spectrometer is 200 to 1100 nm and the resolution is 1.2 nm, according to Jamshidi et al. [25]
- The light source used in this configuration was SLI-CAL (StellarNet, Tampa, FL, USA). The material of this halogen light source is tungsten with a power of 20 W.
- Optical fiber that was used to transmit light from the light source to the samples and from there to the spectrometer according to Nicolai et al. [21]

#### 2.2.2. Pre-Processing of Spectral Data Extracted from Each Sample

After extracting spectral data from each sample, there may be noise for some reasons such as the disturbing ambient light, unevenness of the sample surface, increase in temperature of spectrometer and different sizes of the sample that affects spectral information and causes error. For this reason, raw spectral data must be pre-processed. In this research, preprocessing operations were performed in three stages. The first stage is the conversion of reflectance spectra to absorption spectra (see Equation (1)), the second stage is light scatter and baseline correction using standard normal variant, and the third stage is smoothing using the median filter according to Rossel [26].

Absorption spectra = log (1/eflectance spectra)(1)

### 2.3. Extraction of Chemical Properties of Apple Sample Using Destructive Method

#### 2.3.1. Chemical Properties of TA

Fruits, as they get closer to ripening time, decrease in their acidity and their taste tends to sweeten. Therefore, this feature can be used as an indicator for the ripening time of Gala apples. The method used to destructively measure the acidity of the titration was the method used by James (1998).

#### 2.3.2. Properties of Taste Index

Taste index is defined as the ratio of total soluble solids (TSS) to the titratable acidity (TA). This index is used to determine the taste, which depends on the level of fruit ripening according to Wongkhot et al. [27]) Therefore, this index can be used to determine the ripening stage of Gala apples.

### 2.4. Selection of Key Wavelengths in the Range of 200–1100 nm

Since non-destructive estimation of physicochemical properties such as TA and taste, size and price of device are key elements for the development of portable devices, these elements should be considered. For this reason, instead of using all the spectral information of the 200 to 1100 nm, spectral information related to the key wavelengths should be used. In this study, the hybrid ANN-FA was used to select key wavelengths. The main idea of this algorithm is inspired by the optical connection between fireflies. This algorithm can be considered as a manifestation of swarm intelligence, in which the cooperation (and possibly competition) of simple and low-intelligence members creates a higher level of intelligence that is certainly not achievable by any of the components according to Yang [28] and Ncama et al. [29] The method is that the firefly algorithm sends different vectors of spectral data related to different wavelengths as input to the artificial neural network with the structure shown in Table 1 and the artificial neural network result is recorded as the mean squared error. It should be noted that the output of the artificial neural network is the mentioned chemical properties.

It should be noted that the key wavelength selection operation is performed separately for each chemical property. Any input vector that causes the lowest mean squared error is considered as the optimal vector and the wavelengths within that vector are considered as key wavelengths.

### 2.5. Estimation of Chemical Properties of TA and Taste Index

In this study, the chemical properties of TA and taste index were estimated using hybrid ANN-PSO and PLSR in order to compare the performance of methods based on artificial intelligence and statistics. In order to evaluate the reliability of these methods in different replications, prediction operations were performed for 200 replications.

#### 2.5.1. Hybrid Artificial Neural Network-Particle Swarm Optimization Algorithm (ANN-PSO)

The multilayer perceptron artificial neural network has various adjustable parameters, the optimal adjustment of which ensures its high performance. These parameters include the number of layers, the number of neurons, transfer function, back-propagation network training function and the back-propagation weight/bias learning function. The particle swarm optimization algorithm is a meta-heuristic algorithm that mimics the group movements of birds to optimize various problems. This algorithm was first proposed by Kennedy and Eberhart [30]. Each answer to the problem is considered as a particle. Every particle is constantly searching and moving. The motion of each particle depends on three factors: (1) The current position of the particle, (2) The best position the particle has ever had, and (3) The best position the whole set of particles has ever had according to Kennedy and Eberhart [30]. The number of selectable layers is at least 1 and at most 3, the number of neurons for the first layer is at least 1 and at most 25 and for other layers at least 0 and at most 25. The transfer function was selectable from 13 different functions such as tansig, logsig and purelin. The back-propagation network training function was selectable from 19 different functions such as trainbfg, trainrp and traingd. Finally, the back-propagation weight/bias learning function could be selected from 15 different functions such as learncon, learnh and learnhd. The input of the artificial neural network is the spectral data and the output is the mentioned chemical properties. The method is that the particle swarm optimization algorithm selects different vectors from the structure of the artificial neural network and sends it to the artificial neural network. The error is recorded as the mean squared error. Any structure that results in a minimum MSE is identified as the optimal structure. It should be noted that 60% of the data is randomly used for training, 10% for validation and the other 30% for testing.

#### 2.5.2. Partial Least Squares Regression Method

Partial least squares regression (PLSR) is a statistical method that deals with principal component regression. This is a non-parametric method that is not sensitive to sample size and does not require data normalization according to Rossel [27].

### 2.6. Performance Evaluation of Methods for Estimating the Chemical Properties of TA and Taste Index

In order to evaluate the performance of the hybrid ANN-PSO algorithm and PLSR, the criteria of correlation coefficient (R), coefficient of determination (R2), mean squared error (MSE), root mean squared error (RMSE) and mean absolute error (MAE) were used for Sabzi and Arribas [31].

## 3. Results

### 3.1. Statistical Analysis of Samples

#### 3.1.1. Measured Average of Chemical Properties of Samples

Table 2 gives the measured average of chemical properties of TA and taste index of Gala cultivar in three stages of harvest.

#### 3.1.2. ANOVA Analysis for Property of TA

Since there was no significant difference between the three stages of harvest, regression operations were performed on all 150 samples simultaneously. If there was a significant difference, the regression operation had to be performed separately for each class. The results of ANOVA analysis, Tukey and LSD comparing means are given in Table 3 and Table 4.

#### 3.1.3. ANOVA Analysis for Property of Taste Index

The results of ANOVA analysis, Tukey and LSD comparing means are given in Table 5 and Table 6.

#### 3.1.4. T-Student Test for Property of TA

Test of t-student was performed in order to compare destructive and non-destructive methods for predicting the property of TA based on ANN-PSO and PLSR. The results are given in Table 7 and Table 8.

#### 3.1.5. T-Student Test for Taste Index Property

Test of t-student was performed in order to compare destructive and non-destructive methods for predicting the property of taste index based on ANN-PSO and PLSR. The results are given in Table 9 and Table 10.

### 3.2. Optimal Structure of ANN-PSO Algorithm

#### 3.2.1. Chemical Properties of TA

Table 11 gives the structure of the hidden layers of hybrid ANN-PSO algorithm to estimate the chemical properties of TA. The optimal neural network has three layers with the mentioned properties.

#### 3.2.2. Properties of Taste Index

Table 12 gives the optimal values of the adjustable parameters of the artificial neural network. As can be seen, the optimal structure has two hidden layers.

### 3.3. Key Wavelengths Selected by Hybrid ANN-FA

#### 3.3.1. Chemical Properties of TA

The key wavelengths selected by the hybrid ANN-FA algorithm for TA estimation are 865, 872 and 890 nm.

#### 3.3.2. Chemical Properties of Taste Index

The key wavelengths selected by the hybrid ANN-FA algorithm for taste estimation are 942, 958, 967, 974 and 982 nm.

### 3.4. Performance of Hybrid ANN-PSO Algorithm for Non-Destructive Estimation of Chemical Properties of Gala Apple

#### 3.4.1. Chemical Properties of TA

**Figure 2:**
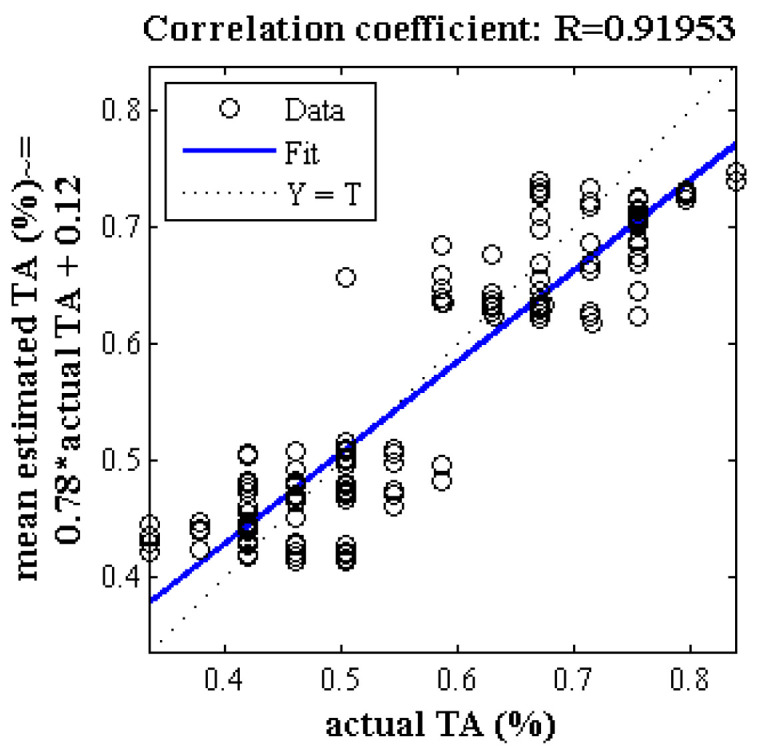
Correlation analysis of a scatter plot between the mean estimated and measured chemical properties of TA of Gala cultivar (test set) by ANN-PSO algorithm based on spectral data related to key wavelengths in 300 replications.

**Figure 3:**
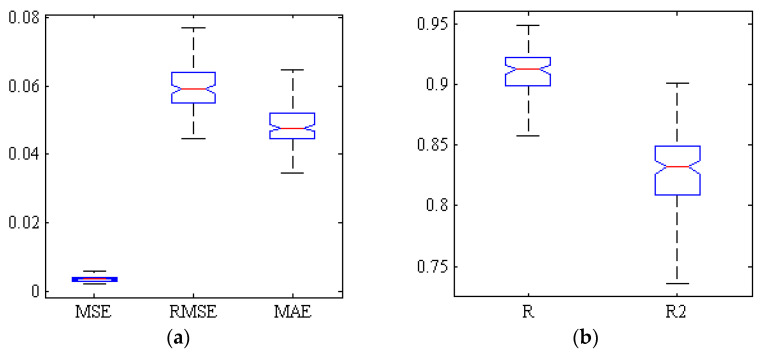
Box of five criteria for evaluating the performance of ANN-PSO algorithm for estimating the TA of Gala cultivar based on spectral data of key wavelengths. (**a**) Error criteria, (**b**) Correlation coefficients and determination.

Figure 2 represents the correlation analysis of the scatter plot between the mean estimated and the measured chemical properties of TA of Gala cultivar (test set) by hybrid ANN-PSO algorithm based on spectral data related to key wavelengths in 300 replications. As can be seen, the correlation coefficient is close to 0.92. Figure 3 shows a box diagram of five criteria for evaluating the performance of ANN-PSO algorithm including R, R2, MSE, MAE and RMSE within the Gala cultivar based on spectral data of key wavelengths in 300 replications. The more compact box diagrams show the similarity of the results in different iterations, which indicate the high validity of the method. As can be seen, the mean squared error in all iterations is less than 0.01 and the value of correlation coefficient in more than half of the iterations is more than 0.91.

#### 3.4.2. Taste Index

**Figure 4:**
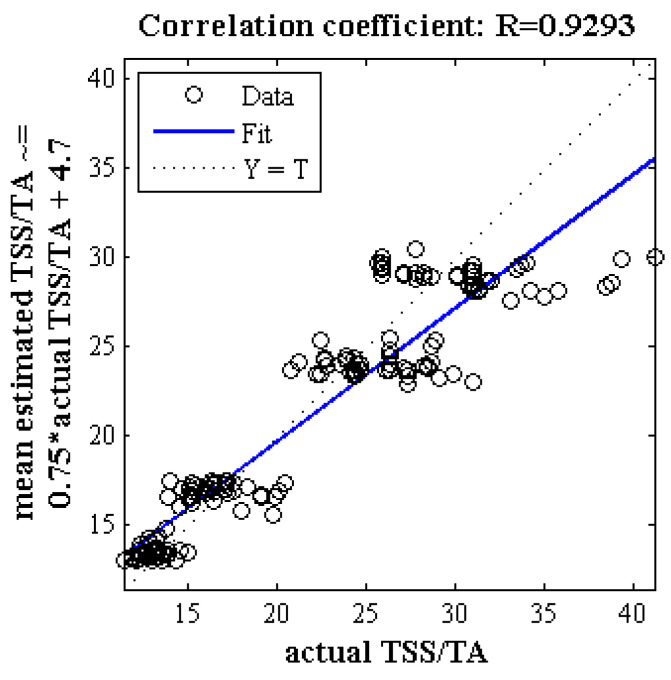
Correlation analysis of scatter plot between mean estimated and measured taste index of Gala cultivar using ANN-PSO algorithm based on spectral data related to key wavelengths in 300 iterations.

**Figure 5:**
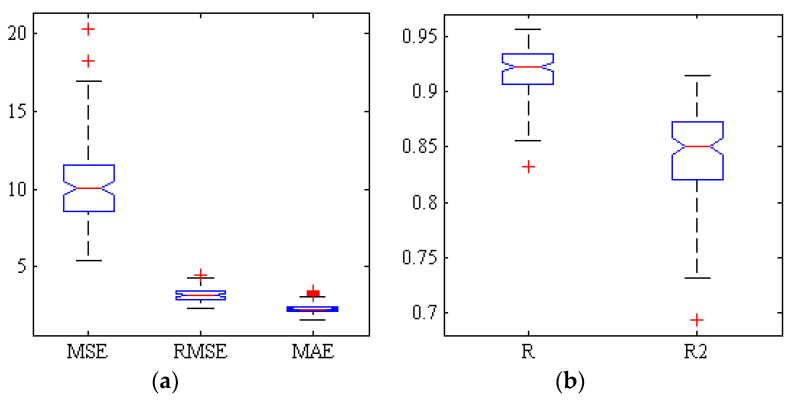
Box of five criteria for evaluating the performance of ANN-PSO for estimating the taste index of Gala cultivar based on spectral data of key wavelengths in 300 iterations. (**a**) Error criteria, (**b**) Correlation coefficients and determination.

Figure 4 illustrates the correlation diagram of the mean estimated and measured properties of taste index of Gala apple in 300 iterations. As can be seen, based on the key wavelength, the correlation coefficient between the mean estimated and measured values is about 0.93. Figure 5 examines the performance of the ANN-PSO for estimating the properties of the taste index using three criteria related to error and correlation coefficients and determination. As can be seen, in more than half of the iterations, RMSE is less than 3. Moreover, except one iteration, the value of correlation coefficient is above 0.85.

## 4. Discussion

### 4.1. Performance of PLSR Method for Non-Destructive Estimation of Chemical Properties of Gala Cultivar

#### 4.1.1. Chemical Properties of TA

**Figure 6:**
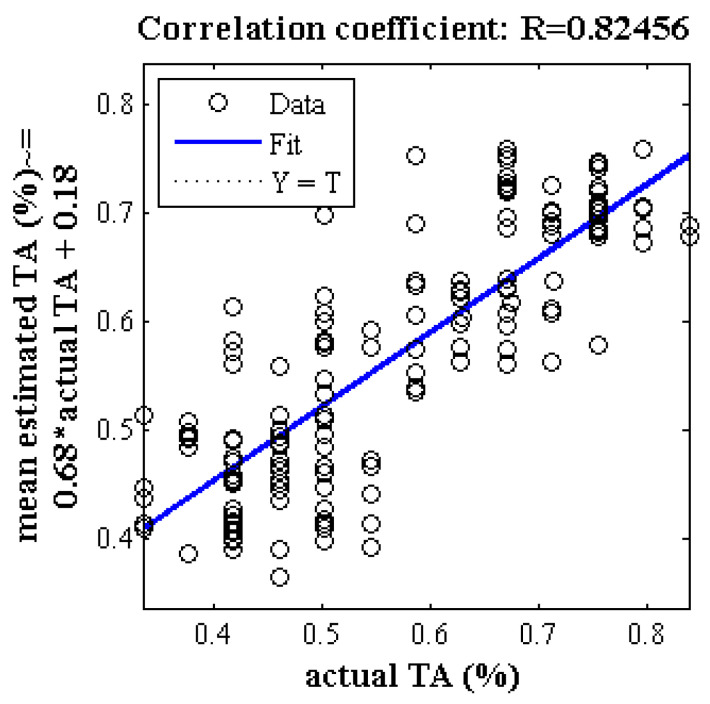
Correlation analysis of scatter plot between mean estimated and measured chemical properties of TA of Gala cultivar using PLSR method based on spectral data related to key wavelengths in 300 iterations.

**Figure 7:**
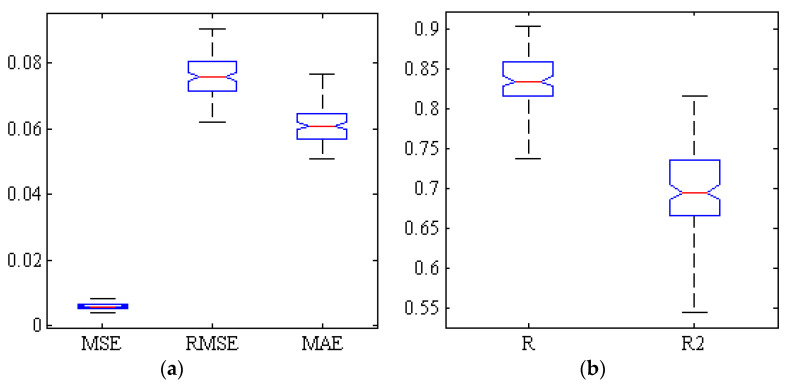
Box diagram of five criteria evaluating the performance of PLSR method for estimating the TA of Gala cultivar based on spectral data of key wavelengths in 300 iterations. (**a**) Error criteria, (**b**) Correlation coefficients and determination.

Figure 6 shows a correlation diagram between the mean estimated and actual value of TA obtained by partial least squares regression method at 300 iterations using the destructive method. The correlation coefficient is slightly more than 0.82, which is lower than the case of ANN-PSO. Figure 7 uses five criteria of mean squared error, root mean squared error, mean absolute error, correlation coefficient and determination coefficient to investigate the performance of PLSR method for estimating the TA. As can be seen, the lowest value of correlation coefficient is above 0.74 and the highest value is close to 0.91.

#### 4.1.2. Chemical Properties of Taste Index

**Figure 8:**
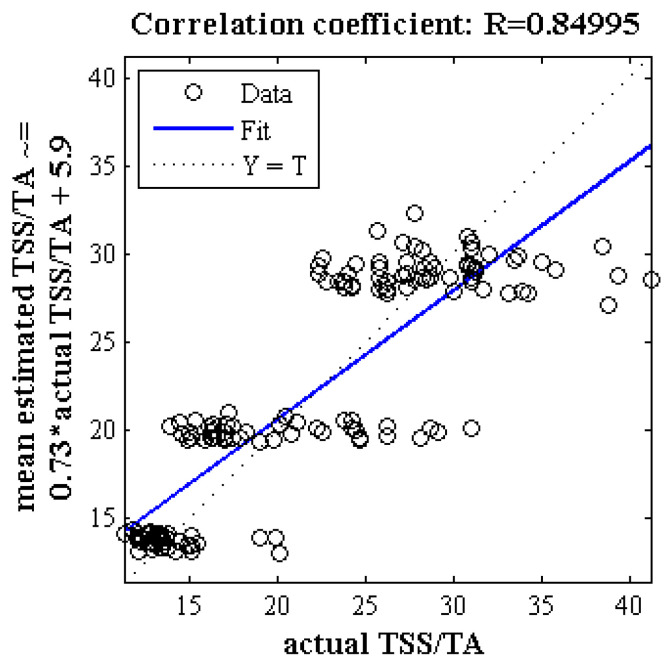
Correlation analysis of scatter plot between mean estimated and measured taste index of Gala apple by PLSR method based on spectral data related to key wavelengths in 300 iterations.

**Figure 9:**
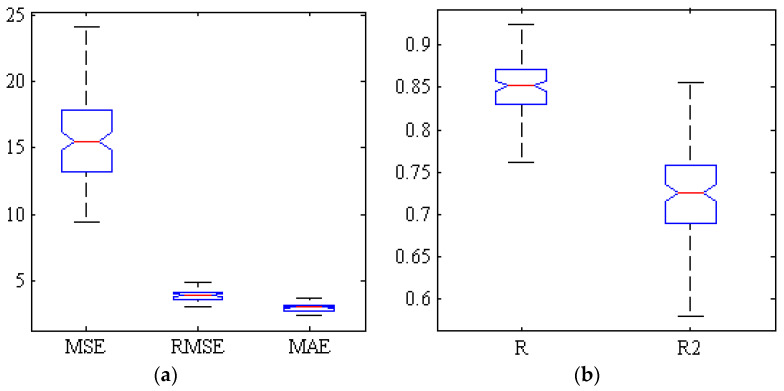
Box diagram of five criteria evaluating the performance of the PLSR method for estimating the taste index based on spectral data of key wavelengths in 300 iterations. (**a**) Error criteria, (**b**) Correlation coefficients and determination.

Figure 8 illustrates the correlation analysis of the scatter plot between the mean estimated and measured taste index of Gala apple (test set) using PLSR method based on spectral data of key wavelengths in 300 iterations. As can be seen, the value of the correlation coefficient in this case is close to 0.85. Figure 9 shows a box diagram of the error criteria and correlation coefficients and determinations used to evaluate the performance of the PLSR method for estimating the taste index of Gala cultivar based on spectral data of key wavelengths at 300 iterations. The lowest coefficient of determination is 0.57 and the highest value is above 0.85.

### 4.2. Comparison of the Performance of Hybrid ANN-PSO Algorithm and PLSR for Non-Destructive Estimation of Chemical Properties of TA

**Figure 10:**
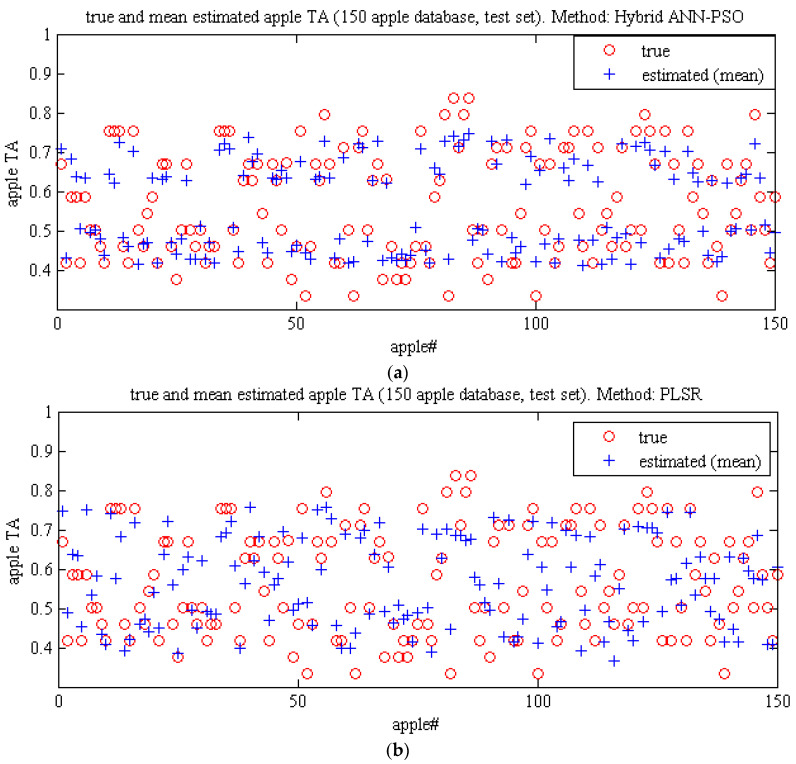
Graphic study of the performance of Methods. (**a**) ANN-PSO Algorithm and (**b**) PLSR for predicting the titratable acidity of Gala apples using spectral data related to key wavelengths.

Figure 10 compares the performance of these two methods using a plot scatter diagram related to the mean estimated and true value of TA using two methods, namely hybrid ANN-PSO algorithm and PLSR. In the ANN-PSO method, the mean estimated values of most samples are closer to the actual values than the PLSR method, which indicates better performance of the ANN-PSO hybrid than the PLSR method. Table 13 compares the mean and standard deviation of different criteria evaluating the performance of the ANN-PSO algorithm and PLSR for estimating the TA at 300 iterations using spectral data of key wavelengths. In the best iteration of ANN-PSO, RMSE and correlation coefficient are 0.0017 and 0.991, respectively. In the case of the PLSR method, these are 0.0040 and 0.9045 respectively. It can be mentioned that the hybrid ANN-PSO method performed better than the PLSR method for estimating TA.

### 4.3. Comparison of the Performance of Hybrid ANN-PSO Algorithm and PLSR for Non-Destructive Estimation of Chemical Properties of Taste Index

**Figure 11:**
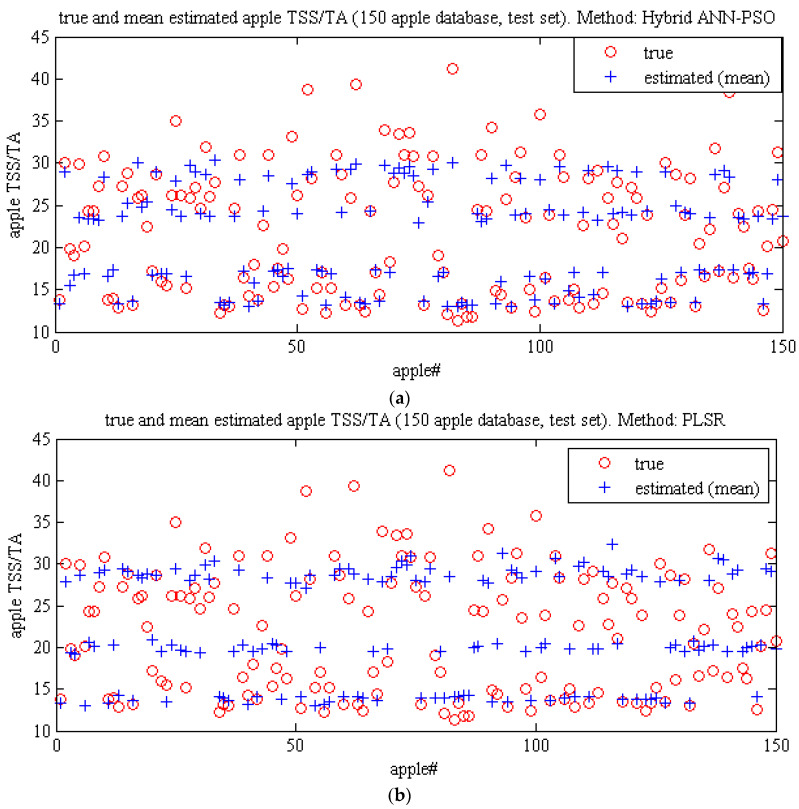
Graphic study of the performance of Methods. (**a**) ANN-PSO Algorithm and (**b**) PLSR for predicting the taste index of Gala apples using spectral data related to key wavelengths.

Figure 11 and Table 14 compare the performance of ANN-PSO methods and partial least squares regression for non-destructive estimation of the taste index value using plot scatter diagrams and various criteria. The ANN-PSO method performs better than the partial least squares regression method.

Table 15 shows the comparison of performance of the proposed algorithm (using methods of hybrid ANN-PSO and PLSR) with other research in the field of non-destructive estimation of TA. As can be seen, the regression coefficient of the proposed algorithm is much higher than other methods.

## 5. Conclusions

In this paper, using a non-destructive method, the chemical properties of the TA and the taste index of the Gala apple at different stages of maturity were estimated. A hybrid ANN-FA method was used to select key wavelengths and two methods, e.g., ANN-PSO and PLSR were used to estimate the mentioned properties. The most important results of this research are listed below:

1. The key wavelengths selected by ANN-FA to estimate the chemical properties of TA and taste index are located in areas outside the range of visible spectra. This is due to the third overtone N-H, the second overtone O-H and the third overtone C-H, which have caused spectral peaks (with useful spectral information) in the range of 870–960 nm [22].
2. Using the hybrid ANN-PSO algorithm, it is possible to estimate with results close to the true value of TA and taste index, e.g., correlation coefficient and RMSE of ANN-PSO for estimating TA and taste index are 0.9491, 0.042, 0.963 and 5.27, respectively. Therefore, since the cost of spectral data extraction is more at higher wavelengths, it can be concluded that it is possible to develop a portable device based on key wavelengths at a low cost for non-destructive estimation of these two properties.
3. The reason for the superiority of the hybrid ANN-PSO algorithm against the PLSR method could be related to the non-linear nature of the artificial neural network and the optimal adjustment of its parameters by the particle swarm optimization algorithm.

## Figures and Tables

**Table 1:** Structure of hidden layers of the neural network used to select key wavelengths.

Features	Layers of the Network
Number of neurons	1st layer: 18
	2nd layer: 6
Number of layers	2
Transfer function	1st layer: tansig
	2nd layer: tansig
Backpropagation Network Training Function	trainrp
Backpropagation Weight/Bias Learning Function	learnk

**Table 2:** Measured average of chemical properties in three stages of harvest.

Chemical Properties	Number of Samples	Measured Average		
		Stage 1	Stage 2	Stage 3
TA	150	0.5557	0.5649	0.5808
Taste Index	150	22.12	22.62	21.24

**Table 3:** ANOVA analysis for property of TA.

TA			Sum of Squares	df	Mean Square	*F*	Sig.
Between Groups	(Combined)		0.016	2	0.008	0.435	0.648
	Linear Term	Contrast	0.016	1	0.016	0.851	0.358
		Deviation	0.000	1	0.000	0.020	0.888
Within Groups			2.713	147	0.018		
Total			2.729	149			

df: the degrees of freedom, *F*: *F*-Value, Sig.: significance.

**Table 4:** Tukey and LSD comparing for property of TA.

TA	(I) Stage	(J) Stage	Mean Difference (I-J)	Std. Error	Sig.
Tukey HSD	1	2	−0.0092189	0.0271709	0.939
		3	−0.0250601	0.0271709	0.627
	2	1	0.0092189	0.0271709	0.939
		3	−0.0158412	0.0271709	0.829
	3	1	0.0250601	0.0271709	0.627
		2	0.0158412	0.0271709	0.829
LSD	1	2	−0.0092189	0.0271709	0.735
		3	−0.0250601	0.0271709	0.358
	2	1	0.0092189	0.0271709	0.735
		3	−0.0158412	0.0271709	0.561
	3	1	0.0250601	0.0271709	0.358
		2	0.0158412	0.0271709	0.561

Std. Error: Standard Error, Sig.: significance.

**Table 5:** ANOVA analysis for property of taste index.

Taste Index			Sum of Squares	df	Mean Square	*F*	Sig.
Between Groups	(Combined)		48.580	2	24.290	0.430	0.651
	Linear Term	Contrast	19.248	1	19.248	0.341	0.560
		Deviation	29.332	1	29.332	0.519	0.472
Within Groups			8304.080	147	56.490		
Total			8352.660	149			

df: the degrees of freedom, *F*: *F*-Value, Sig: significance.

**Table 6:** Tukey and LSD comparing for property of taste index.

Taste Index	(I) Stage	(J) Stage	Mean Difference (I–J)	Std. Error	Sig.
Tukey HSD	1	2	−0.4993301	1.5032011	0.941
		3	0.8774570	1.5032011	0.829
	2	1	0.4993301	1.5032011	0.941
		3	1.3767872	1.5032011	0.631
	3	1	−0.8774570	1.5032011	0.829
		2	−1.3767872	1.5032011	0.631
LSD	1	2	−0.4993301	1.5032011	0.740
		3	0.8774570	1.5032011	0.560
	2	1	0.4993301	1.5032011	0.740
		3	1.3767872	1.5032011	0.361
	3	1	−0.8774570	1.5032011	0.560
		2	−1.3767872	1.5032011	0.361

Std. Error: Standard Error, Sig.: significance.

**Table 7:** T-Student test for TA property based on PLSR Method.

	Levene’s Test for Equality of Variances			*t*-Test for Equality of Means					
	*F*	Sig.	*t*	df	Sig. (2-Tailed)	Mean Difference	Std. Error Difference	95% Confidence Interval of the Difference	
								Lower	Upper
Equal variances assumed	11.134	0.001	−0.011	298	0.991	−0.00015396	0.01434931	−0.02839278	0.02808485
Equal variances not assumed			−0.011	288.025	0.991	−0.00015396	0.01434931	−0.02839677	0.02808884

*F*: *F*-Value, Sig.: significance, *t*: *t*-test statistic, df: the degrees of freedom, Std. Error: Standard Error.

**Table 8:** T-Student test for TA property based on ANN-PSO Method.

	Levene’s Test for Equality of Variances		*t*-Test for Equality of Means						
	*F*	Sig.	*t*	df	Sig. (2-Tailed)	Mean Difference	Std. Error Difference	95% Confidence Interval of the Difference	
								Lower	Upper
Equal variances assumed	4.026	0.046	0.540	298	0.590	0.0078267	0.0144987	−0.0207061	0.0363594
Equal variances not assumed			0.540	290.399	0.590	0.0078267	0.0144987	−0.0207091	0.0363624

*F*: *F*-Value, Sig.: significance, *t*: *t*-test statistic, df: the degrees of freedom, Std. Error: Standard Error.

**Table 9:** T-Student test for Taste Index property based on PLSR Method.

	Levene’s Test for Equality of Variances		*t*-Test for Equality of Means						
	*F*	Sig.	*t*	df	Sig. (2-Tailed)	Mean Difference	Std. Error Difference	95% Confidence Interval of the Difference	
								Lower	Upper
Equal variances assumed	6.709	0.010	0.002	298	0.999	0.0013963	0.8007309	−1.5744073	1.5771999
Equal variances not assumed			0.002	290.032	0.999	0.0013963	0.8007309	−1.5745838	1.5773765

*F*: *F*-Value, Sig.: significance, *t*: *t*-test statistic, df: the degrees of freedom, Std. Error: Standard Error.

**Table 10:** T-Student test for Taste Index property based on ANN-PSO Method.

	Levene’s Test for Equality of Variances		*t*-Test for Equality of Means						
	*F*	Sig.	*t*	df	Sig. (2-Tailed)	Mean Difference	Std. Error Difference	95% Confidence Interval of the Difference	
								Lower	Upper
Equal variances assumed	9.016	0.003	1.020	298	0.309	0.8002578	0.7846944	−0.7439865	2.3445022
Equal variances not assumed			1.020	284.964	0.309	0.8002578	0.7846944	−0.7442746	2.3447903

*F*: *F*-Value, Sig.: Significance, *t*: *t*-test statistic, df: the degrees of freedom, Std. Error: Standard Error.

**Table 11:** Structure of hidden layers of ANN-PSO algorithm to estimate TA.

Features	Layers of the Network
Number of neurons	1st layer: 19
	2nd layer: 13
	3nd layer: 16
Number of layers	3
Transfer function	1st layer: tribas
	2nd layer: poslin
	3nd layer: tansig
Backpropagation Network Training Function	trainbr
Backpropagation Weight/Bias Learning Function	learngd

**Table 12:** The structure of the hidden layers of ANN-PSO algorithm to estimate the taste index.

Features	Layers of the Network
Number of neurons	1st layer: 21
	2nd layer: 9
Number of layers	2
Transfer function	1st layer: radbas
	2nd layer: tansig
Backpropagation Network Training Function	traingd
Backpropagation Weight/Bias Learning Function	learnk

**Table 13:** Comparison of mean and standard deviation, different criteria evaluating the performance of the ANN-PSO algorithm and PLSR for estimating TA in 300 iterations using spectral data of key wavelengths.

Method	Criteria	MSE	RMSE	MAE	*R*	*R* ^2^
Hybrid ANN-PSO	Mean and standard deviation in 300 iterations	0.0036 ± 0.0008	0.0598 ± 0.0064	0.0486 ± 0.0054	0.9095 ± 0.0175	0.8274 ± 0.0316
	In the best state of training	0.0017	0.0420	0.0382	0.09491	0.09008
PLSR	Mean and standard deviation in 300 iterations	0.0058 ± 0.0009	0.0761 ± 0.0061	0.0612 ± 0.0055	0.8340 ± 0.0313	0.6965 ± 0.0519
	In the best state of training	0.0040	0.0631	0.0523	0.9045	0.8181

**Table 14:** Comparison of mean and standard deviation, different criteria evaluating the performance of the ANN-PSO algorithm and PLSR for estimating the taste index in 300 iterations using spectral data of key wavelengths.

Method	Criteria	MSE	RMSE	MAE	*R*	*R* ^2^
Hybrid ANN-PSO	Mean and standard deviation in 300 iterations	10.41 ± 2.63	3.20 ± 0.39	2.31 ± 0.33	0.918 ± 0.02	0.844 ± 0.04
	In the best state of training	5.27	2.29	1.73	0.963	0.927
PLSR	Mean and standard deviation in 300 iterations	16.96 ± 3.33	4.09 ± 0.403	3.17 ± 0.316	0.836 ± 0.033	0.699 ± 0.055
	In the best state of training	9.82	3.13	2.94	0.923	0.852

**Table 15:** Comparison of the regression coefficient, R, of the proposed method with other research for the estimation of the TA of Gala apples.

*R*	No. of Samples	Fruit	Property	Research Work
0.963	150	Apple	TA	Proposed method (ANN-PSO)
0.923	150	Apple	TA	Proposed method (PLSR)
0.53	333	Apple	TA	Liu and Ying [32]
0.53	200	Apple	TA	Ignat et al. [33]
0.91	960	Apple	TA	Lan et al. [24]
